# Cardiac Phenotypes in Fabry Disease: Genetic Variability and Clinical Severity Staging Correlation in a Reference Center Cohort

**DOI:** 10.3390/genes16091086

**Published:** 2025-09-15

**Authors:** Denise Cristiana Faro, Serena Di Salvo, Margherita Stefania Rodolico, Valentina Losi, Davide Capodanno, Ines Paola Monte

**Affiliations:** 1 Department of General Surgery and Medical-Surgical Specialties, University of Catania, Via S. Sofia 78, 95123 Catania, Italy; denisecristiana.faro@unict.it (D.C.F.); serenads47@gmail.com (S.D.S.); vale.losi@gmail.com (V.L.); dcapodanno@unict.it (D.C.); 2 Institute for Biomedical Research and Innovation, National Research Council (IRIB-CNR), Section of Catania, Via Paolo Gaifami 18, 95126 Catania, Italy; margheritastefania.rodolico@cnr.it; 3 Cardiology Unit—CAST, AOU Policlinico “G. Rodolico-S. Marco”, Via S. Sofia 76, 95123 Catania, Italy

**Keywords:** Anderson-Fabry disease, clinical outcomes, rare diseases, lysosomal storage, X-linked, stratification, prognosis, heart failure, arrhythmias

## Abstract

Background/Objectives: Anderson–Fabry disease (AFD) presents with a wide spectrum of clinical manifestations, influenced by the underlying *GLA* genotype. While classical variants are typically associated with early-onset, multisystemic involvement, late-onset mutations and variants of uncertain significance (VUS) often display predominantly cardiac phenotypes. This study aimed to explore the relationship between *GLA* variant class, cardiovascular severity, and clinical outcomes using validated staging systems and real-world data. Methods: In this single-centre retrospective study, we evaluated 42 patients with genetically confirmed AFD, stratified into classical, late-onset, and VUS categories. Cardiovascular involvement was assessed using three standardized staging tools—Del Franco, Meucci, and MSSI—and correlated with the occurrence and burden of major adverse cardiovascular events (MACE). Multivariable analyses were performed to adjust for age, sex, and treatment status. Results: Classical variants were strongly associated with more advanced cardiac staging (Del Franco, Meucci) and higher MSSI scores, reflecting systemic disease severity. These patients experienced significantly more frequent and severe MACE (*p* = 0.022), confirming the prognostic relevance of genotypic stratification. In contrast, carriers of late-onset mutations and VUS exhibited milder phenotypes and lower event rates. Importantly, genotype remained an independent predictor of cardiovascular risk in adjusted models, suggesting a direct contribution to disease progression beyond demographic or therapeutic factors. Conclusions: This study highlights the role of *GLA* genotype in shaping cardiovascular risk and clinical trajectory in AFD. Integrating genetic classification with clinical staging provides a powerful, multimodal approach to risk stratification and supports the move toward genotype-informed, personalized management strategies in AFD.

## 1. Introduction

Anderson-Fabry disease (AFD, OMIM #301500) is a rare X-linked lysosomal storage disorder caused by mutations in the *GLA* gene (Gene Entrez: 2717; NCBI reference sequence: NM_000169.3; OMIM #300644; Locus Reference Genomic record LRG_672) located on chromosome Xq22.1, which encodes the lysosomal hydrolase α-galactosidase A (α-Gal A, EC 3.2.1.22; Uniprot P06280) [1].

The enzyme deficiency leads to progressive accumulation of glycosphingolipids—especially globotriaosylceramide (Gb3) and its deacylated derivative lyso-Gb3—within lysosomes of various cell types, including vascular endothelium, cardiomyocytes, renal podocytes, and neurons [2]. The result is a multisystemic disorder characterized by inflammation, oxidative stress, mitochondrial dysfunction, and eventually irreversible fibrosis [2,3,4,5].

Although historically considered rare, with an incidence of 1:40,000 to 1:117,000, neonatal screening programs have revealed a much higher prevalence, especially of late-onset variants with cardiac or renal involvement. Some studies suggest a frequency as high as 1 in 3100 live births and 1:8882 in Italian reports [6,7,8,9]. This discrepancy highlights both the clinical and genetic heterogeneity of AFD, and the need for careful classification of *GLA*-variants [10,11], especially in patients with non-classical phenotypes.

### 1.1. The Genetic Landscape of GLA Variants

To date, more than 1000 variants in the *GLA* gene have been identified, encompassing missense, nonsense, splicing, frameshift, and large genomic rearrangements [12]. The interpretation of these variants relies on multiple criteria, including predicted impact on protein structure, enzymatic activity, family segregation, and biochemical evidence. According to ACMG guidelines, *GLA* variants are categorized as benign, likely benign, variants of uncertain significance (VUS), likely pathogenic, or pathogenic [13].

Classical AFD is typically associated with null variants (e.g., nonsense or frameshift mutations), which lead to undetectable α-Gal A activity (<1%) and early-onset multiorgan disease in hemizygous males. In contrast, missense mutations—such as the well-known p.N215S variant—may retain residual enzymatic activity and are often linked to cardiac-predominant phenotypes with later onset and slower progression [14,15].

In female heterozygotes, clinical expression is highly variable due to X-chromosome inactivation (lyonization), resulting in a mosaic distribution of α-Gal A activity. Some females remain asymptomatic throughout life, while others develop substantial cardiac or renal involvement. This variability underscores the importance of genetic testing for diagnosis and risk assessment, particularly in women with borderline enzymatic activity [16,17].

The metabolic and pathologic phenotypes represent distinct manifestations of substrate accumulation, assessed by biochemical and histological methods. Along with the enzymatic phenotype, they serve as specific diagnostic markers of AFD, being direct consequences of enzyme deficiency. In contrast, variants’ clinical phenotypes are more distantly related to the genetic cause and are shaped by factors such as gender, age, other modifier genetic loci, polygenic traits, epigenetic factors, and environmental risks, for example, Cardiometabolic risk factors (smoking, hypertension, and obesity) [15,18]

A key challenge is distinguishing AFD’s nonspecific late-onset manifestations—especially cerebrovascular, cardiac, and renal—from similar common conditions. Comprehensive phenotyping, ideally in hemizygous males, is essential to assess variant severity, clarify the effects of hypomorphic alleles, define benign polymorphisms, and determine the pathogenicity of uncertain variants.

Moreover, de novo mutations have been described, and the absence of a positive family history does not exclude the disease. In this context, genotype-phenotype correlation remains an area of active investigation, with potential implications for therapeutic stratification and early intervention.

### 1.2. Cardiac Involvement and Its Clinical Impact

Cardiac manifestations represent the leading cause of morbidity and mortality in Fabry patients and often precede or dominate the clinical picture, especially in non-classical variants. Gb3 accumulation in cardiomyocytes triggers cellular hypertrophy, energy imbalance, and chronic inflammation, leading to concentric left ventricular hypertrophy (LVH), diastolic dysfunction, atrial enlargement, arrhythmias, and myocardial fibrosis. In advanced stages, Fabry cardiomyopathy mimics hypertrophic or restrictive phenotypes, and systolic dysfunction may eventually develop [19,20].

The inflammatory component of Fabry cardiomyopathy has gained increasing recognition. Gb3 and lyso-Gb3 can act as damage-associated molecular patterns (DAMPs), activating innate immune receptors such as Toll-like receptor 4 (TLR4) and stimulating pro-inflammatory pathways involving IL-1β, TNF-α, and reactive oxygen species [2]. These mechanisms contribute not only to myocardial remodelling and fibrosis, but also to coronary microvascular dysfunction and electrophysiological instability [21].

Given the high burden of cardiac disease in AFD and its prognostic relevance, several imaging and clinical markers have been proposed to monitor progression and guide treatment [22,23,24]. However, stratifying patients remains a challenge due to the heterogeneity of presentation and the absence of genotype integration in most risk models.

### 1.3. Toward a Structured Stratification: Three Complementary Models

In recent years, objective staging criteria for cardiac involvement in AFD have been proposed, including the clinical-imaging classification by Del Franco et al. (DF-Stad), the echocardiographic model by Meucci et al. (ME-Stad), and the multidomain Mainz Severity Score Index (MSSI).

Del Franco et al. outlined four stages of Fabry cardiomyopathy, from a non-hypertrophic phase with subtle ECG or imaging changes, through hypertrophic stages—with or without myocardial fibrosis—up to overt ventricular dysfunction and symptomatic heart failure [25]. Cardiac magnetic resonance (CMR), particularly late gadolinium enhancement (LGE), plays a central role in identifying irreversible fibrosis.

Meucci et al. proposed a simplified echocardiographic staging system based on left ventricular wall thickness, left atrial size, and ventricular function [26]. Their four-stage model correlated progressively with adverse cardiovascular outcomes, highlighting the importance of diastolic markers such as E/e′ and TAPSE in disease progression [27].

The MSSI, while broader and less cardiac-specific, integrates systemic, neurological, renal, and cardiovascular features into a cumulative score to quantify overall disease burden [28]. It remains useful for long-term follow-up and therapy response monitoring, especially under enzyme replacement or chaperone therapy.

These systems are complementary: imaging-based models offer detailed cardiac phenotyping, while MSSI captures systemic severity—particularly relevant in multi-organ involvement. However, none currently incorporates genotype-based stratification, despite growing evidence of variant-specific risk profiles.

### 1.4. What Is Missing: Knowledge Gap and Challenges in Stratification and Aims of the Study

Despite considerable progress in the understanding of AFD and growing evidence that *GLA* variant type influences disease phenotype, genotype–phenotype correlation is still a main challenge of current research: a knowledge gap persists regarding the relationship between the type of *GLA* gene variant and the severity of clinical manifestations, particularly cardiac involvement [29] and few studies have examined whether this stratification correlates with cardiovascular outcomes or structured clinical/imaging staging systems [30,31,32].

Some cross-sectional investigations have reported differences in phenotype severity and sex-related expression patterns [17,33], together with micro and microvascular involvement and complications, also by capillaroscopic patterns, correlated with variant subgroups [34]. N215S has been linked to elevated LVMI and fibrosis [14,15], and others have highlighted genotype-phenotype associations for specific mutations such as F113L or A143T [10,35,36].

However, the impact of variant class on cardiac staging systems—such as DF-Stad or ME-Stad—and on global severity scores like MSSI remains unexplored. This gap is particularly relevant in the era of precision medicine and could inform more personalized follow-up and management strategies (e.g., initiation of ERT or migalastat) and risk prediction algorithms [37,38].

We report the first such comprehensive analysis, strengthening the genetic basis for risk stratification and personalized therapy.

Our study aims to bridge this gap by investigating genotype–phenotype correlations in Anderson-Fabry disease with a specific focus on cardiac involvement. Specifically, we sought to characterize cardiac manifestations in a cohort of genetically confirmed AFD patients by integrating multimodal cardiac imaging and classifying individuals according to established staging systems (Del Franco, Meucci, and MSSI).

We further aimed to assess the relationship between GLA variant class (classical, late-onset, and VUS) and disease severity as expressed by these structured staging systems, and to evaluate the impact and predictive value of genotype class on the occurrence of major adverse cardiac events (MACE).

By combining phenotypic stratification with genetic data, this study provides a comprehensive overview of disease expression and contributes to the refinement of risk stratification in Fabry patients, with the ultimate goal of supporting more personalized management strategies.

## 2. Materials and Methods

### 2.1. Study Design and Population

We conducted a retrospective, observational study including consecutive patients with a confirmed genetic diagnosis of AFD evaluated between January 2014 and February 2025 at the Rare Cardiomyopathies and Echocardiography Clinic of the “G. Rodolico” University Hospital in Catania, Italy. Patients aged ≥ 18 years with available comprehensive clinical, laboratory, and instrumental data (ECG, echocardiography, and, when available, cardiac MRI) were eligible (Table 1). Clinical, genetic, and imaging data were collected from electronic medical records in our dedicated AFD database and systematically reviewed.

Patients underwent a thorough anamnesis and physical examination, with construction of family pedigree, and a systematic assessment of cardiac and extracardiac involvement based on standard protocols, searching for AFD typical red flags at the screening time, and to grade the severity of organ involvement at each follow-up:

- Cardiac: ECG, echocardiogram; CMR and Holter ECG were prescribed to all patients but not everyone accepted to undergo these examinations;
- Neurological: MRI, MR angiography, electromyography, visual evoked potentials, autonomic function tests;
- Renal: serum creatinine, urine analysis, renal biopsy if indicated by the specialist;
- Audiological: audiometry, brainstem auditory evoked potentials;
- Pulmonary: chest X-ray/CT scan, spirometry;
- Gastrointestinal: abdominal ultrasound, CT if needed;
- Ophthalmologic: slit-lamp and fundus examination;
- Vascular: Doppler ultrasound, nailfold capillaroscopy;
- Dermatologic: clinical identification of angiokeratomas;
- Psychological issues and quality of life.

Follow-up visits are generally scheduled on an annual basis, with additional evaluations at 3 to 6 months if required due to pathological conditions requiring closer monitoring, or if the patient reports symptoms or a worsening of exercise tolerance.

### 2.2. Clinical Endpoints

The primary clinical endpoint was the occurrence of major adverse cardiovascular events (MACE), defined as the composite of cardiovascular death, ischemic stroke, arrhythmias (atrial fibrillation-AF, major ventricular arrhythmias, bradyarrhythmias requiring pacing, pacemaker [PM]/implantable cardioverter defibrillator [ICD] implantation and appropriate ICD shocks), worsening HF (to NYHA 3–4), HF hospitalization, MINOCA (myocardial infarction with non-obstructive coronary arteries), vascular thrombo-embolic events, over a period from first diagnosis to the latest follow-up (Table 1).

### 2.3. Cardiac Staging and Classification

Cardiac staging was performed using two recently validated staging models—DF-Stad and ME-Stad—based on clinical features together with ECG, echocardiographic, and cardiac magnetic resonance (CMR) parameters (Table 2). Each patient was also assessed using the MSSI to capture systemic disease burden.

A 12-derivation standard ECG with attention to AFD red flags (brady-tachi arrhythmias, PR interval, atrio-ventricular or intraventricular conduction defects, delta wave, LVH signs, ST/T alterations, QT interval) was performed at each visit (CardioSoft v6.7 Diagnostic System from GE Healthcare, Milwaukee, WI)

Comprehensive standard and advanced transthoracic echocardiography was performed using a Vivid-E95 system (GE, Norway) following EACVI/ASE guidelines [39] and latest consensus papers about the integration of multimodal CV imaging [24], including right and left ventricular diameter, volumes, and thickness, systolic and diastolic function, and aortic root. We also applied speckle-tracking echocardiography (STE) to evaluate longitudinal strain (GLS). Analyses were conducted offline using EchoPAC v2.02 (GE Healthcare, Chicago, IL, USA).

CMR was performed at multiple centres using local protocols, according to patient consent and collaboration. Assessed parameters included wall thickness, LVEF, myocardial mass, cine imaging sequences, and LGE. T1 mapping and extracellular volume (ECV) were available in selected cases.

Arrhythmias were assessed through 24–48 h Holter monitoring and/or device interrogation, and included ventricular fibrillation (VF), sustained (>30 s) and non-sustained (<30 s) ventricular tachycardia (VT), atrial fibrillation (AF), and supraventricular tachycardias.

Exertional dyspnoea was defined according to NYHA classification.

Cardiac involvement was classified according to the clinical-imaging staging system proposed by Del Franco et al. [25], which stratifies Fabry cardiomyopathy into four stages based on ECG, echocardiography, and cardiac magnetic resonance (CMR) findings, with particular emphasis on the detection of myocardial fibrosis via late gadolinium enhancement (LGE).

Patients were also staged using the echocardiographic classification proposed by Meucci et al. [26], which assesses disease severity across four progressive stages based on echo standard parameters: left ventricular wall thickness (LVWT), left atrial volume index (LAVi), ejection fraction (LVEF), E/e′ ratio, and tricuspid annular plane systolic excursion (TAPSE).

Both systems were used to assess correlations between cardiac staging and genotype class, clinical presentation, and outcomes.

The MSSI is a validated tool developed to quantify AFD severity, track disease progression, and monitor treatment efficacy. It is based on four domains:

1. General: systemic symptoms such as fever, fatigue, and weight loss.
2. Neurological: includes acroparesthesias, hypohidrosis, and other neurological signs.
3. Cardiac: assesses arrhythmias, ventricular hypertrophy, and other cardiac findings.
4. Renal: evaluates renal function via proteinuria and serum creatinine.

The total score stratifies disease severity into Mild 0–20; Moderate: 21–40; Severe: >40 [28].

### 2.4. Genetic Analysis and Multidisciplinary Management

Genetic testing was performed via polymerase chain reaction (PCR) amplification and full sequencing of the *GLA* gene. All samples were processed at Institute for Biomedical Research and Innovation (IRIB-CNR, Palermo, Italy). Alongside genotyping, enzymatic activity of α-galactosidase A and plasma levels of lyso-Gb3 were measured in accordance with international diagnostic standards [22,38,40]. Variant interpretation followed ACMG guidelines, and variants were classified as pathogenic, likely pathogenic, of uncertain significance (VUS), likely benign, or benign [13].

This process was conducted as part of the initial diagnostic workup for AFD and integrated within the institutional diagnostic protocol. All patients provided specific additional written informed consent for the genetic analysis.

### 2.5. Ethical Considerations

The study was conducted in accordance with the principles of the Declaration of Helsinki and received approval from the Ethics Committee of the University Hospital “G. Rodolico—San Marco” of Catania, Italy. All patients signed informed consent authorizing the use of their clinical, imaging, and genetic data in anonymized and aggregated form for scientific research purposes.

Additionally, a dedicated informed consent for genetic testing and associated biochemical analyses (α-Gal A enzymatic activity, lyso-Gb3 quantification, and *GLA* variant detection) was obtained from each subject prior to testing. Genetic counselling was offered where appropriate, particularly in cases involving variants of uncertain significance or implications for familial screening.

### 2.6. Statistical Analysis

Continuous variables were expressed as mean ± SD or median with interquartile range (IQR), as appropriate (Kolmogorov-Smirnov test for normality). Categorical variables were reported as absolute and relative frequencies.

Chi-square or Fisher’s exact test was used to assess the association between genotype class and binary outcomes. Logistic regression was applied to evaluate odds ratios (OR) with 95% confidence intervals (CI) for the association between genotype class and the occurrence of MACE. Kruskal–Wallis and Wilcoxon rank-sum tests were employed for non-parametric comparisons of MACE counts and clinical staging distributions (DF-Stad, ME-Stad, MSSI) across variant groups. Ordinal logistic regression with logit link was used to assess associations between genotype classes and clinical staging systems. For independent evaluation of each genotype class (Classical, Late-Onset, VUS), regression models were also fitted without assuming any reference group. Since MSSI is a continuous score, linear regression models were applied for each genotype independently, reporting β coefficients with 95% CIs. As a sensitivity analysis, all genotype–phenotype associations were re-evaluated in the subgroup of patients with available CMR data, with descriptive comparisons reported due to limited sample size. A *p*-value < 0.05 was considered statistically significant. Statistical analyses were performed using SPSS ver.26 and Python 3.11.

## 3. Results

### 3.1. Patient Characteristics

A total of 65 patients with genetically confirmed AFD were included (Table 3). The mean age at diagnosis was 40.6 ± 15.1 years, with a female predominance (75.4%). Classical mutations were present in 21.5% of cases, late-onset variants in 36.9%, and VUS in 28.5%. Benign polymorphisms accounted for 3.1% of the cohort, but were not included in the statistical analyses.

Most patients were untreated at baseline (75.4%), while 18.5% received enzyme replacement therapy (ERT) and 6.2% were on migalastat. Regarding cardiovascular medications, 21.5% were taking beta-blockers, 10.8% calcium channel blockers, 9.2% ACE inhibitors, 10.8% angiotensin receptor blockers, 12.3% diuretics, 4.6% antiarrhythmics, 15.4% antiplatelet agents, and 3.1% oral anticoagulants. Direct oral anticoagulants (DOACs) were used in one patient (1.5%, apixaban), while 7.7% had undergone dialysis or renal transplantation. The mean baseline values of α-Gal A activity and LysoGb3 were 7.35 ± 5.21 nmol/mL/h and 6.13 ±14.19 nmol/L, respectively. The median Mainz Severity Score Index (MSSI) was 10.0 [IQR: 5– 14].

Comorbidities included hypertension (44.6%), dyslipidaemia (18.5%), cerebral ischemic vasculopathy (13.8%), and chronic kidney disease (16.9%). Median serum creatinine was 0.71 [0.6–0.9] mg/dL, with proteinuria in 6.2%.

Echocardiography showed a median maximal left ventricular (LV) wall thickness of 8 [7–10.5] mm and an indexed LV mass (LVMi) of 70 [58–91] g/m^2^; 17% met criteria for LV hypertrophy. The median ejection fraction was 65% [61–66.5], and global longitudinal strain (GLS) was mildly reduced in some patients (median −18.5% [IQR: −21 to −16]). The left atrial volume index (LAVI) was 25 [20–31] mL/m^2^, and TAPSE was 22 [20–31] mm.

Most patients were in sinus rhythm (96.9%); one had atrial fibrillation, one had a pacemaker, and one an ICD. CMR was performed in 20%, revealing late gadolinium enhancement in 13.8% and abnormal native T1 in a minority of cases.

### 3.2. Clinical Outcomes and Cardiovascular Events

In a timeframe for capturing the events (from the time of first diagnosis to latest FU visit) of 60,7 months (median), a total of 25 MACE were recorded among 12 patients (18.5%) (Table 4). These included:

- Cardiovascular death (*n* = 2, 3%);
- Stroke, transient ischemic attack (TIA), or arterial thromboembolism (*n* = 11, 16.9%);
- Atrial fibrillation (AF) (*n* = 3, 4.5%);
- Progression to NYHA class III–IV (*n* = 1, 1.5%);
- Hospitalization for HF (*n* = 1, 1.5%);
- Myocardial infarction with non-obstructive coronary arteries (MINOCA) (*n* = 1, 1.5%);
- Device implantation (1 PM and 1 ICD) (*n* = 2, 3%);
- Non-sustained ventricular tachycardias (NSVT) (*n* = 4, 6%), although none progressed to sustained arrhythmia or required ICD intervention.

In addition, one death due to a work-related accident occurred and was not considered related to cardiovascular disease.

### 3.3. Cardiac Staging and Risk Stratification 

Applying the DF-Stad, 41.5% of patients were classified in stage 0 (non-hypertrophic with early markers), 4.6.% in stage 1 (LVH without fibrosis), 12.3% in stage 2 (LVH with fibrosis), and 41.5% in stage 3 (overt dysfunction) (Table 5).

Notably, patients in stages 2–3 showed significantly higher MSSI scores (*p* < 0.01) and greater incidence of LGE on CMR.

Using the ME-Stad, 80% of patients were in stage 0, 6.2% in stage 1, 10.8% in stage 2, and 3.1% in stage 3.

There was substantial concordance between the two classifications, especially in identifying advanced disease. Advanced stages in both systems correlated with GLS impairment, LA enlargement, and elevated LVMi.

MSSI showed a median value of 10.0 [IQR 5; 14], with only one patient over 40 pt, 4 patients in the moderate range, and most patients (60) under 20 pt.

### 3.4. Arrhythmic Burden and Conduction Abnormalities

Holter ECG monitoring revealed non-sustained ventricular tachycardia (NSVT) in 4.6%, and only one patient had permanent AF. No sustained ventricular arrhythmias or sudden cardiac deaths were recorded. Pacemaker implantation was necessary in a single patient due to high-grade AV block with syncope.

### 3.5. Multiorgan Involvement and MSSI Correlation

Higher MSSI scores were significantly associated with female sex, older age, and classical phenotypes. MSSI also correlated with worse renal function (*p* = 0.02), LVH (*p* < 0.01), and echocardiographic stage (*p* < 0.01). Moreover, MSSI ≥ 15 was predictive of advanced cardiac involvement and higher risk of arrhythmia (OR: 2.8, 95% CI: 1.1–7.4).

### 3.6. Genetics

#### 3.6.1. Genetic Mutations in the *GLA* Gene

**Figure 1:**
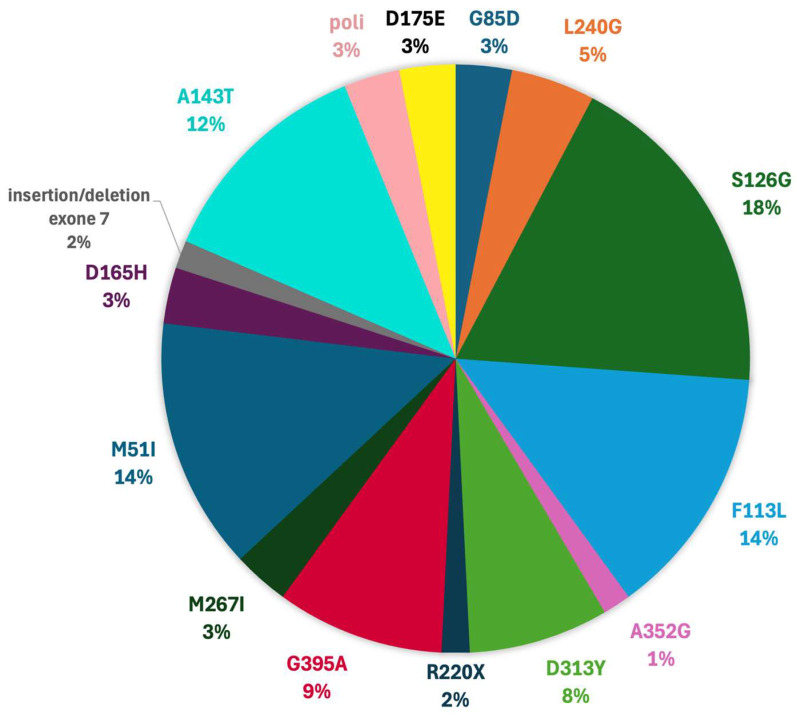
*GLA* mutations distribution in our population.

The mutational landscape observed in our cohort was notably heterogeneous, encompassing missense, nonsense, intronic, and structural variants of the *GLA* gene. These mutations were associated with a spectrum of clinical phenotypes ranging from classical AFD to late-onset variants and VUS (Figure 1).

Among the most frequently encountered variants, S126G emerged as the most prevalent. This missense mutation involves the substitution of serine with glycine at position 126 and is typically classified as a VUS, although its high frequency in the cohort suggests possible pathogenic relevance. F113L, another commonly observed mutation, is well-documented in the literature and often linked to late-onset cardiac phenotypes. The A143T variant, while frequent, remains controversial: some studies support a pathogenic role, while others suggest a more benign or uncertain classification.

The M51I and G395A variants were also recurrently observed, both associated with variable phenotypic expression. Less frequent mutations included G85D, L240G, M267I, D165H, D175E, and A352G, some of which (e.g., G85D and D165H) are strongly associated with classical Fabry phenotypes.

We also identified intronic mutations such as IVS4 + 6A > C, which may impact splicing and gene expression, and structural alterations like insertion/deletion in exon 7, potentially leading to significant functional disruption. The D313Y variant, although historically considered benign or a VUS, has recently been associated with hypertrophic cardiomyopathy, particularly when coexisting with mutations in other cardiomyopathy-related genes (e.g., MYH7).

Overall, the most represented pathogenic or likely pathogenic variants in our dataset were S126G, F113L, A143T, M51I, and G395A. Their phenotypic variability underscores the necessity of integrating genetic data with clinical and imaging findings to accurately stratify patient risk and guide personalized management strategies.

#### 3.6.2. Genotype–Phenotype Correlation and Outcome Analysis

Pathogenic mutations were significantly more frequent in patients with advanced echocardiographic or CMR-based stages (*p* = 0.03). F113L and A143T variants were primarily found in patients with isolated cardiac involvement, whereas classical mutations like D165H were more commonly associated with multiorgan disease and higher MSSI scores. Over a median follow-up of 3.5 years, MACE occurred in 6.2%, predominantly in those with LVH, LGE, or GLS > −15%. No deaths were recorded during follow-up.

#### 3.6.3. Association Between Genotype Class and Composite Cardiovascular Events

To evaluate the prognostic impact of *GLA*-variant classification on clinical outcomes, we first investigated the association between genotype class (Classical, Late-Onset, VUS) and the occurrence of MACE, defined as a composite of cardiac death, myocardial infarction, stroke, heart failure, arrhythmias, and NYHA class worsening.

**Figure 2:**
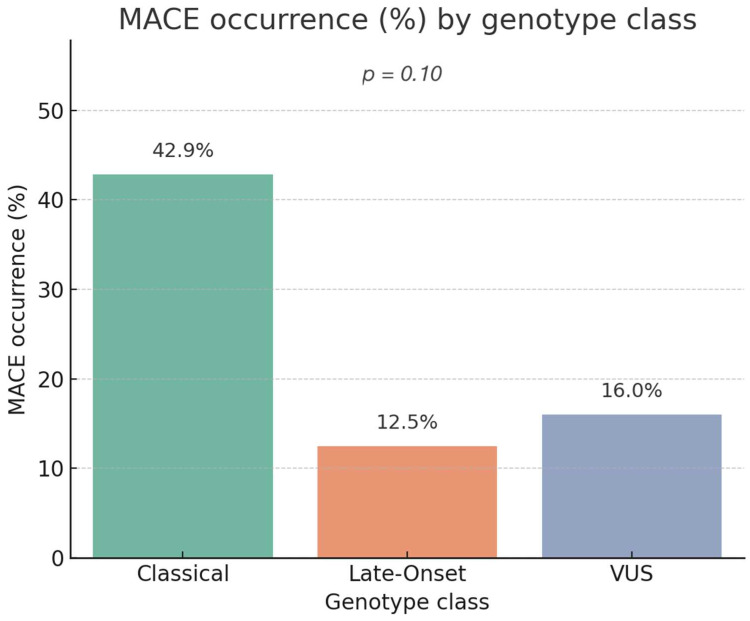
MACE occurrence (%) by genotype class. The proportion of patients experiencing major adverse cardiac events (MACE) is shown for each genotype category: Classical, Late-Onset, and Variants of Uncertain Significance (VUS). Percentages are displayed above each bar. Statistical comparison among groups was performed using the chi-square test (*p* = 0.10). Abbreviations: MACE = major adverse cardiac events; VUS = variant of uncertain significance.

The global chi-square test comparing MACE incidence across variant groups showed a trend toward statistical significance (χ^2^ = 6.17, *p* = 0.10), suggesting a potential but not definitive association between genotype and clinical events (Figure 2).

To better quantify risk, separate logistic regressions were conducted for each genotype class. Carriers of classical variants had a significantly higher risk of MACE (OR: 4.50; 95% CI: 1.19–16.96; *p* = 0.026), confirming the strong prognostic weight of classical mutations. In contrast, patients with Late-Onset variants (OR: 0.44; 95% CI: 0.11–1.80; *p* = 0.256) and VUS (OR: 0.66; 95% CI: 0.18–2.41; *p* = 0.526) showed no statistically significant association with increased cardiovascular risk. (Table 6). These results support the utility of genotypic classification in early risk stratification.

#### 3.6.4. Association Between Genotype Class and Burden (Number) of MACE

**Figure 3:**
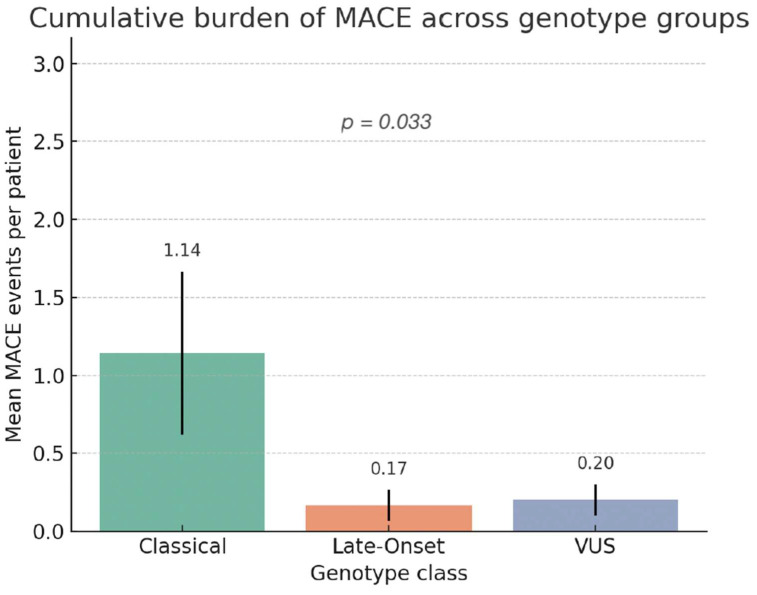
Cumulative burden of MACE across genotype groups. The mean number of major adverse cardiac events (MACE) per patient is shown for Classical, Late-Onset, and Variants of Uncertain Significance (VUS) genotypes. Bars represent mean values with standard error of the mean (SEM). Statistical comparison among groups was performed using the Kruskal–Wallis test (*p* = 0.033). Abbreviations: MACE = major adverse cardiac events; VUS = variant of uncertain significance.

In a subsequent analysis, we evaluated the cumulative burden of cardiovascular events across genotype groups. The Kruskal–Wallis test demonstrated a significant difference in MACE counts among genotypes (χ^2^ = 6.82, *p* = 0.033) (Figure 3). Classical variant carriers exhibited the highest median number of events (2.0 [IQR 1–3]), significantly higher than both Late-Onset (0.0 [0–1], *p* = 0.015) and VUS (0.0 [0–0], *p* = 0.048). No significant difference was observed between Late-Onset and VUS groups (*p* = 0.41).

These findings reinforce the substantial clinical burden associated with classical variants and highlight the relative benignity of other genotypes.

#### 3.6.5. Stratified Analyses by Sex and Therapy

**Figure 4:**
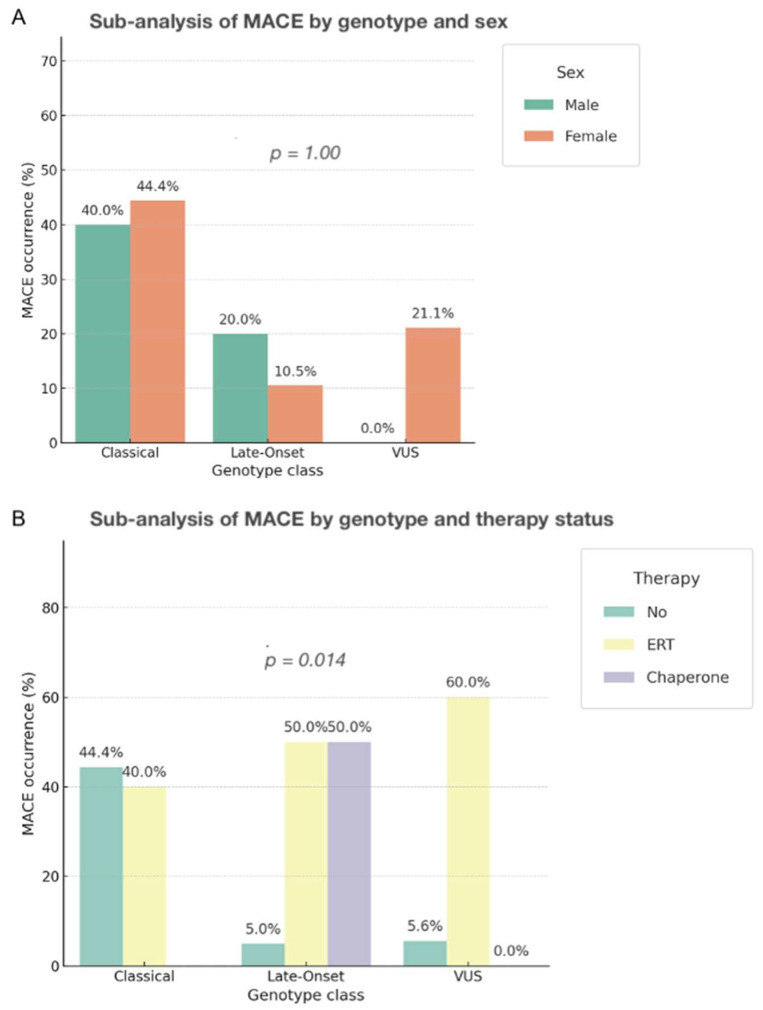
Subgroup analysis of MACE by genotype class according to sex and therapy status. (**A**) The proportion of patients experiencing major adverse cardiac events (MACE) is shown for each genotype, stratified by sex (male vs. female). No significant difference was observed between sexes (*p* = 1.00). (**B**) When stratified by therapy status, patients receiving enzyme replacement therapy (ERT) showed higher proportions of MACE compared with untreated individuals. The overall chi-square test indicated a statistically significant association (χ^2^ = 8.61, *p* = 0.013). However, this result should be interpreted with caution: confidence intervals were wide (e.g., ERT OR 2.32, 95% CI 0.53–4.11; Chaperone OR 3.03, 95% CI −0.47–6.53) and included unity, indicating lack of robust evidence. This pattern likely reflects confounding by indication, since more severely affected patients were preferentially treated, leading to an apparent but not causal association. Abbreviations: MACE = major adverse cardiac events; ERT = enzyme replacement therapy; VUS = variant of uncertain significance.

Exploratory stratified analyses showed that the association between classical genotype and MACE remained consistent across sex and treatment subgroups, though significance was attenuated in smaller strata. No clear interaction effects emerged. Treated vs. untreated subgroup comparisons showed a non-significant trend toward fewer events in treated individuals, independent of genotype (Figure 4).

#### 3.6.6. Association Between Genotype Class and Clinical Staging (ME-Stad, DF-Stad, MSSI)

We then examined how genotype classification related to three staging systems of cardiac involvement: the DF-Stad (0–3, echo-based), the ME-Stad (0–3, integrated), and the MSSI (continuous).

**Figure 5:**
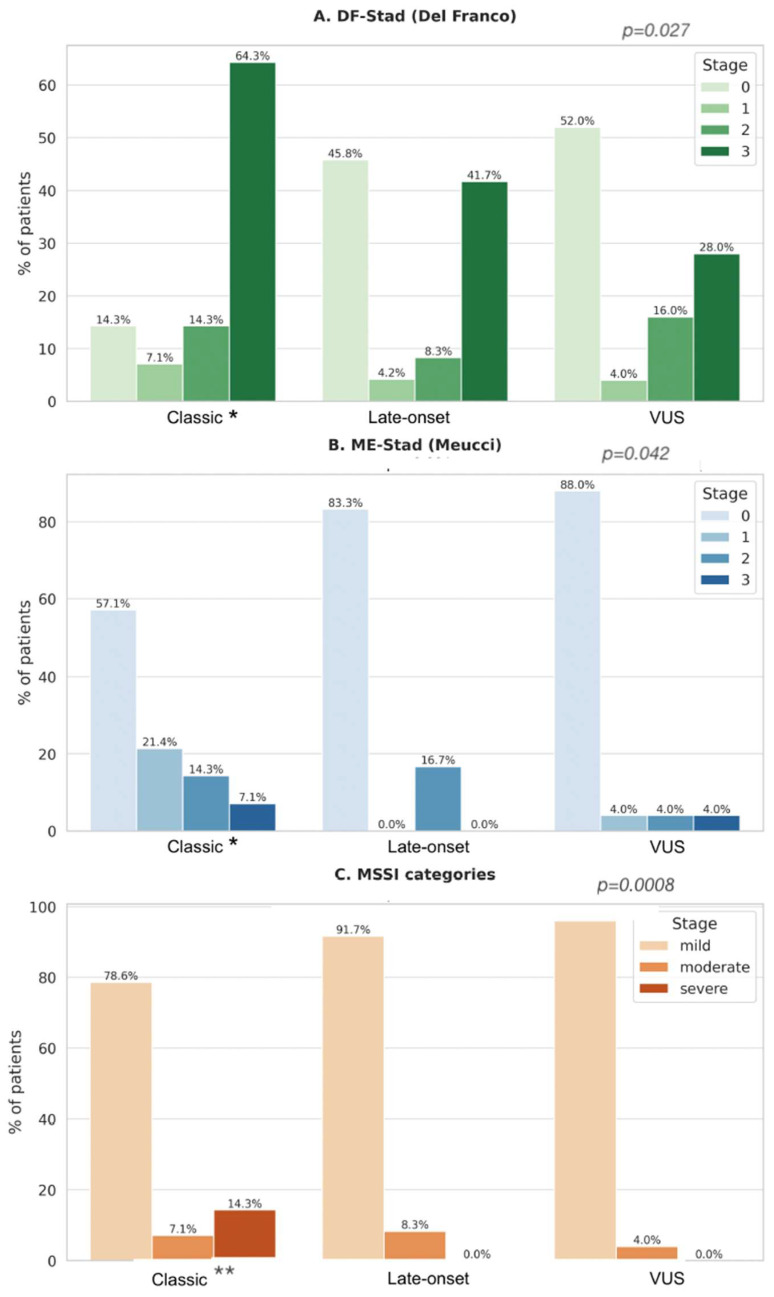
Distribution of clinical staging systems by genotype class. Barplots showing the distribution of patients across genotype classes (Classical, Late-Onset, VUS) for the three staging systems: (**A**) DF-Stad (Del Franco, 0–3), (**B**) ME-Stad (Meucci, 0–3), and (**C**) MSSI categories (mild, moderate, severe). Percentages are shown above each bar. Panel headers report model-based *p*-values: ordinal logistic regression (logit) for DF-Stad and ME-Stad testing Classical vs. others (two-sided Wald tests), and linear regression for MSSI testing Classical vs. others Model *p*-values: DF-Stad (ordinal logistic, Classical vs. others) *p* = 0.027; ME-Stad (ordinal logistic, Classical vs. others) *p* = 0.042; MSSI (linear, Classical vs. others) *p* = 0.0008. Polymorphisms excluded. (two-sided *t*-test). As for ordinal regression results (next to the genotype class: * *p* < 0.05, ** *p* < 0.001). Abbreviations: DF-Stad = Del Franco staging; ME-Stad = Meucci staging; MSSI = Mainz Severity Score Index; VUS = variant of uncertain significance.

Descriptively, classical variant carriers were more frequently classified at advanced stages in all three systems (Figure 5A–C):

- DF-Stad: 85% of classical carriers were in stage ≥ 2 vs. 29% of Late-Onset and 25% of VUS.
- ME-Stad: 80% of classical carriers reached stage ≥ 2 vs. 29% of Late-Onset and 12.5% of VUS.
- MSSI score: Classical carriers had a median MSSI of 24, significantly higher than Late-Onset [12] and VUS [8].

To further quantify these associations, regression models were applied.

Genotype class was related to disease severity across staging systems. In ordinal logistic models (treating genotype class as independent that classical variants were significantly associated with higher staging severity both in ME-Stad (OR 3.80, 95% CI 1.05–13.7, *p* = 0.041) and DF-Stad (OR 3.84, 95% CI 1.17–12.6, *p* = 0.027), whereas Late-Onset and VUS showed no significant associations. (Table 7), Treating MSSI as a continuous outcome, linear regression confirmed higher scores in Classical variant carriers (β = +9.11; 95% CI 4.08–14.15; *p* = 0.0008), with non-significant differences for Late-Onset and VUS.

Non-parametric omnibus comparisons across genotype classes showed a significant difference for MSSI (Kruskal–Wallis χ^2^ = 8.90, *p* = 0.0116), a borderline effect for DF-Stad (χ^2^ = 5.80, *p* = 0.055), and no difference for ME-Stad (χ^2^ = 4.82, *p* = 0.090). This pattern accords with the model-based analyses, in which greater severity is driven primarily by Classical carriers.

These results demonstrate a robust relationship between genotype class and disease severity, with classical mutations driving higher risk both in terms of outcomes and cardiac damage extent.

#### 3.6.7. Sensitivity Analysis in Patients with Available CMR Data

To evaluate whether the limited availability of CMR data influenced the robustness of our findings, we performed a sensitivity analysis restricted to the 13 patients who underwent CMR. In this subgroup, classical variants were associated with more advanced cardiac involvement, with a median Del Franco stage of 3.0, higher mean MSSI scores (21.9), and fibrosis detected by LGE in 62.5% of cases. MACE occurred in 50% of classical carriers, with a mean of 3.25 events per affected patient (Table 8). Late-onset variants (*n* = 3) showed comparable Del Franco stages (median 3.0), but with lower MSSI values (mean 16.0), a lower proportion of MACE (33.3%), and LGE positivity in 67%. VUS carriers (*n* = 2) presented with lower Meucci and MSSI scores (mean 0.0 and 15.0, respectively), but both patients experienced MACE and exhibited LGE positivity (100%). Although formal statistical testing was not feasible due to the small sample size and sparse group distribution, these descriptive findings paralleled the associations observed in the overall cohort, reinforcing the robustness of the main results.

The table reports mean and median Del Franco stage, mean Meucci stage, mean Mainz Severity Score Index (MSSI), frequency of major adverse cardiac events (MACE, %), mean number of MACE per affected patient, and prevalence of late gadolinium enhancement (LGE, %). Despite limited sample size precluding formal statistical testing, descriptive results paralleled the overall cohort, with classical variants showing higher severity and VUS displaying heterogeneous but clinically relevant involvement.

## 4. Discussion

### 4.1. Genotype, Cardiac Involvement, and Staging Severity

In this study, we investigated the association between *GLA* genotype class and cardiovascular involvement in patients with AFD, using both clinical outcomes and validated staging systems.

Our findings demonstrate that even in the earliest stages of disease, subtle structural and functional cardiac abnormalities are present and possess predictive value for adverse cardiovascular outcomes.

Classical variants were significantly associated with advanced cardiac staging across all three systems adopted in this study—DF-Stad, ME-Stad, and MSSI—underscoring their prognostic weight. The DF-Stad enabled the identification of two distinct phenotypes: early-stage patients (stages 0–1), characterized by preserved function and absence of fibrosis, and more advanced-stage patients (stages 2–3), exhibiting LVH, atrial enlargement, and LGE on CMR. The ME-Stad added key echocardiographic insights, such as left atrial volume and diastolic function, complementing the staging assessment. Notably, classical variant carriers showed the highest scores across all systems, while late-onset variants and VUS displayed milder phenotypes.

### 4.2. Genotype–MACE Association and Risk Stratification

A clear association between genotype class and major adverse cardiovascular events (MACE) emerged in our cohort. Classical variants conferred a significantly increased risk of experiencing MACE (*p* = 0.022), as well as a greater event burden, compared to late-onset or VUS carriers. Although the global chi-square for genotype distribution did not reach conventional significance (*p* = 0.10), the trend supports the hypothesis that *GLA* variant class can guide cardiovascular risk stratification. Importantly, VUS and late-onset mutations were not significantly associated with MACE occurrence or burden, suggesting a comparatively milder cardiac phenotype.

To our knowledge, no prior study has simultaneously assessed the correlation between *GLA* variant type, cardiovascular staging systems, and clinical outcomes in a well-characterized cohort with longitudinal follow-up. This integrative approach offers a more complete picture of the prognostic value of genotypic stratification in Fabry cardiomyopathy. Several studies have previously addressed genotype-related clinical variability in AFD and documented increased morbidity in classical males [10,16]. McCarron et al. reported that patients with the N215S variant showed increased cardiovascular morbidity and mortality, with a higher MSSI and earlier complications, while Lavalle et al. demonstrated later symptom onset, development of LVH, proteinuria, and occurrence of cerebrovascular events, with an overall severity by MSSI comparable to the non-N215S despite lower lyso-Gb3 levels [14,15].

Similarly, observational studies on F113L and IVS4 variants demonstrated a strong cardiac predilection, with AF, ventricular arrhythmias, and conduction disturbances, and reports of myocardial fibrosis preceding hypertrophy in F113L families in Portugal [35,41,42]. Although these late-onset mutations are traditionally considered cardiac-limited, they are still associated with measurable disease burden—especially in hemizygous males or in presence of comorbidities. Our data confirm milder staging scores and event burden in these late-onset carriers, consistent with their residual enzyme activity and predominantly cardiac-limited phenotype.

Some case reports and a recent meta-analysis have raised questions about the pathogenicity of VUS such as A143T and D313Y (also referred to as A313T), describing phenotypes with cardiomyopathy with LVH [43,44,45] and arrhythmias [46]. However, other studies report these variants as non-disease-causing [47], highlighting persistent discrepancies in pathogenicity classification between sources such as the ClinVar database and ACMG criteria. These conflicting interpretations affect genotype-phenotype correlations, with significant implications for therapeutic decision-making [48,49].

While our group previously described sex-based differences and variant prevalence in cardiac and vascular disease manifestations and complications, those works did not assess outcome-driven stratification [17,34]. Moreover, few studies have evaluated genotype effects on composite endpoints including both structural parameters and clinical events.

Our study results refine and expand upon these findings by integrating genotypic classification with structured staging models and event-based outcome analysis, lacking in earlier studies.

### 4.3. Staging Systems and Independent Prognostic Role of Genotype

Importantly, the relationship between genotype and disease severity persisted even after adjusting for age, sex, and therapy in multivariable models, supporting the concept that *GLA* variant class independently influences prognosis and reinforcing the importance of incorporating genotype into staging frameworks and risk prediction models.

Our data show that classical variants were associated with worse outcomes and more advanced stages across all three classification systems, suggesting that early genotype identification may allow for timely and personalized management.

VUS and late-onset variants showed significantly lower association with advanced staging and MACE: their residual enzymatic activity likely explains the milder phenotype. Thus, our study confirms that VUS, though often ambiguous, did not confer elevated cardiovascular risk in our cohort in terms of major adverse outcomes and severity of cardiac involvement.

Nonetheless, the absence of statistical significance should not be misinterpreted as complete benignity—especially in older individuals or those with cardiovascular comorbidities: as the absence of significant findings in VUS and late-onset groups may reflect smaller sample sizes or reduced penetrance, these variants should not be dismissed as clinically irrelevant.

As shown before, in our cohort, carriers of A143T and D313Y variants displayed milder phenotypes without a clear correlation to major adverse cardiac events. These findings are consistent with recent consensus statements, which emphasize that VUS should not be regarded as pathogenic unless supported by functional assays, segregation data, or strong biochemical evidence [10,40,50,51].

Importantly, VUS carriers should not be dismissed as unaffected: periodic follow-up with clinical assessment, cardiac imaging, and monitoring of lyso-Gb3 levels is advised, as phenotypic expression may evolve over time [17]. Recent studies have further illustrated how reinterpretation of individual variants can substantially change classification and clinical implications [48,52]. More recent recommendations further specify that VUS alone should not trigger initiation of disease-specific therapy, such as enzyme replacement therapy or chaperone therapy, in the absence of convincing clinical or biochemical evidence. Instead, longitudinal monitoring, systematic re-evaluation and biomarker assessment, and periodic variant reclassification according to updated ACMG/ClinGen frameworks are advocated, and in selected cases of VUS carriers with early organ involvement, individualized treatment decisions may be considered after thorough multidisciplinary evaluation [49,53,54]. Collectively, these data underscore the need for a cautious but proactive approach, ensuring adequate surveillance of VUS carriers while avoiding premature or unnecessary therapeutic interventions.

### 4.4. Sex-Related Differences and Their Clinical Implications in Fabry Disease

In our cohort, which included a majority of female patients (75%), we observed pronounced sex-related variability in the degree of cardiac involvement. This heterogeneity is consistent with the X-linked inheritance of Fabry disease and the biological consequences of random X-chromosome inactivation (lyonization), which creates a mosaic distribution of α-galactosidase A activity across tissues [55,56]. As a result, women carrying *GLA* variants cannot be considered unaffected: they may range from being nearly asymptomatic to developing severe cardiomyopathy and major adverse cardiac events [17,57,58].

Our sex-stratified analysis confirmed this variability. While men carrying classical variants consistently showed more advanced staging and adverse outcomes, a substantial proportion of women with the same variants also exhibited LGE-defined fibrosis and clinical events. Conversely, women with late-onset or VUS variants generally displayed milder phenotypes, although relevant organ manifestations were occasionally present. These findings are in line with recent systematic reviews and consensus recommendations underscoring the broad phenotypic heterogeneity among female patients and the need for individualized monitoring strategies [16,33]. 

Importantly, current expert opinions emphasize that treatment decisions in female patients should not be delayed or withheld solely on the basis of sex, but instead be guided by objective evidence of organ involvement documented by imaging, biomarkers, or clinical presentation [40]. Taken together, our findings reinforce that genotype–phenotype correlations in Fabry disease must always be interpreted in the context of sex-specific expression and lyonization, and that women require tailored risk stratification and structured longitudinal follow-up.

### 4.5. Summary and Translational Relevance

The integration of genotype-based classification with clinical staging frameworks such as those proposed by Meucci and Del Franco offers a robust model for phenotype stratification in AFD.

Our data suggest that genotype does not simply predict the presence or absence of cardiac involvement but rather modulates the trajectory of structural and functional cardiac remodelling. This is evident in the differential staging profiles observed across variant classes: classical variants were more frequently staged in advanced disease (DF-Stad 2–3, ME-Stad 3), with concordantly higher MSSI scores, while late-onset variants and VUS were predominantly staged in the lower categories, often with preserved function.

This nuanced stratification has important implications for both research and clinical care. From a research standpoint, our findings validate the utility of integrating genotype in disease modelling, staging validation, and outcome prediction. From a clinical perspective, the results advocate for incorporating genotype-based risk profiles into decision algorithms, including those guiding therapy initiation and intensity of follow-up. In particular, patients harbouring classical *GLA* variants may benefit from earlier cardiac imaging, shorter intervals between follow-up visits, and earlier access to disease-specific therapies, even in the absence of overt cardiomyopathy. Furthermore, this study reinforces the idea that genetic stratification should not be limited to diagnostic confirmation but rather employed dynamically throughout the care pathway. As new therapies targeting substrate reduction, gene therapy, and pharmacological chaperoning emerge, personalized medicine in AFD will increasingly rely on precise genotype–phenotype correlations to guide therapeutic choices.

### 4.6. Study Limitations and Future Directions

Our study is limited by its retrospective design and the monocentric nature of the cohort, which may affect generalizability, particularly since the population is predominantly Italian and Caucasian. Nevertheless, the consistency of our findings with other European Fabry cohorts supports their external validity. Moreover, only 20% of patients underwent cardiac magnetic resonance (CMR), despite late gadolinium enhancement (LGE) being a central element in DF-Stad staging. This limited use of CMR may have reduced staging accuracy, as fibrosis could have been underdetected in patients assessed solely with echocardiography and clinical criteria. However, echocardiographic and clinical staging remain widely applied in real-world settings.

A sensitivity analysis restricted to patients with available CMR data confirmed the robustness of our findings. Although statistical comparisons were not feasible due to the small sample size and sparse genotype distribution, descriptive results paralleled the overall cohort, with classical variants associated with higher staging, MSSI scores, and LGE-defined fibrosis, while VUS showed heterogeneous but clinically relevant cardiac involvement. These findings support a staged model of disease where genotype drives phenotypic progression through cardiac fibrosis, diastolic dysfunction, and hypertrophy.

Lastly, LysoGb3 levels and α-galactosidase A activity were not included in follow-up or regression analyses, as these data were not consistently available across the cohort. In addition, follow-up measurements were non-standardized and referred to a heterogeneous patient population (with or without treatment, receiving different drug classes), making their inclusion beyond the scope of this study.

Despite these limitations, it however remains among the first to directly correlate *GLA* genotype classes with both structured cardiac staging and clinical outcomes.

The integration of genetic class with multimodal staging (DF-Stad + Meucci + MSSI) enhances prognostication beyond what clinical imaging alone can provide. This stratified model is in line with personalized medicine and pharmacogenomic principles and supports timely identification of patients who would benefit most from early treatment initiation.

In the future, prospective multicenter studies incorporating lyso-Gb3 levels, CMR T1 mapping, and even polygenic risk scores will be essential to refine and validate this risk stratification model.

## 5. Conclusions

This study highlights the prognostic role of *GLA* genotype classification in the phenotypic expression and clinical progression of Anderson–Fabry disease. By integrating genotypic data with standardized cardiac staging systems and composite cardiovascular outcomes, we demonstrate that classical variants confer a markedly higher burden of disease and risk of major adverse cardiovascular events. These findings support the clinical utility of a comprehensive model for risk stratification that incorporates genetic variant classification into routine assessment, enabling earlier identification of high-risk patients and more tailored therapeutic strategies.

## Figures and Tables

**Table 1:** Inclusion criteria and clinical endpoints.

Inclusion Criteria	Clinical Endpoints (MACE)
Age ≥ 18 years Confirmed genetic diagnosis of AFD Signed informed consent	Cardiovascular death Heart failure hospitalization Worsening HF (NYHA 3–4)
Availability of ECG, echocardiography, and (when available) CMR	Arrhythmias (AF, major VAs, bradyarrhythmias requiring pacing, e.g., AVBs)
Sufficient clinical and laboratory data for staging Optimal acoustic window for echo images analysis	Device implantation (ICD, PM) Appropriate device interventions MINOCA Stroke
	Vascular Thromboembolic events

Abbreviations: AFD = Anderson-Fabry disease; MACE = Major adverse cardiovascular events; HF = Heart failure; NYHA = New York Heart Association class; CMR = Cardiac magnetic resonance; AF = Atrial fibrillation; VAs = Ventricular arrhythmias; AVBs = Atrioventricular blocks; ICD = Implantable cardioverter defibrillator; PM = Pacemaker; MINOCA = Myocardial infarction with non-obstructive coronary arteries.

**Table 2:** Del Franco e Meucci staging parameters.

**Del Franco**	
Stage 0 (Non-hypertrophic)	LV wall thickness (LVWT) ≤ 12 mm; normal systolic/diastolic function; absence of fibrosis or imaging abnormalities.
Stage 1 (Hypertrophic, pre-fibrotic)	LV wall thickness (LVWT) ≤ 12 mm; normal systolic/diastolic function; absence of fibrosis or imaging abnormalities.
Stage 2 (Hypertrophic, fibrotic)	LVWT > 12 mm; evidence of myocardial fibrosis on CMR (LGE positive); possibly altered LAVi and E/e′.
Stage 3 (Overt dysfunction)	Reduced LVEF < 50%, E/e′ ≥ 15, LAVi > 34 mL/m^2^, TAPSE < 17 mm, extensive LGE, and advanced clinical symptoms.
**Meucci**	
Stage 0	No structural/functional cardiac involvement;
Stage 1	Isolated LVH
Stage 2	Presence of left atrial dilation
Stage 3	Overt ventricular dysfunction (LVEF < 50%, E/e′ ≥ 15, or TAPSE < 17 mm).

**Table 3:** (A) and (B) General and Cardiovascular characteristics of the Study Population (*n* = 65).

(A) Demographic and Clinical	
Parameter	AFD *n* = 65
Age at diagnosis (years)	40.6 ± 15.1
Sex	
-Male	16 (24.6%)
-Female	49 (75.4%)
*GLA* variant	
-Classic	14 (21.5%)
-Late onset	24 (36.9%)
-VUS	25 (28.5%)
-Polymorphism	2 (3.1%)
Baseline specific therapy	
-No	49 (75.4%)
-ERT	12 (18.5%)
-Migalastat	4 (6.2%)
BMI (kg/m^2^)	24.2 [21; 27]
A-Gal -A (nmol/mL/h)	7.35 ± 5.21
Lyso-Gb3 (nmol/L)	6.13 ± 14.19
NYHA-baseline I	43 (66.2%)
NYHA-baseline II	22 (33.8%)
Syncope	5 (7.7%)
Comorbidities	
Hypertension	29 (44.6%)
Diabetes	7 (10.8%)
Cerebral ischemic disease	9 (13.8%)
Coronary and peripheral vasculopathy	11 (16.9%)
Dyslipidaemia	12 (18.5%)
Smoking history	10 (15.4%)
Creatinine (mg/dL)	0.71 [0.6; 0.9]
Proteinuria	4 (6.2%)
Chronic kidney disease	4 (6.2%)
Dialysis	5 (7.7%)
Kidney transplant	5 (7.7%)
(B) Cardiac Imaging Parameters	
Parameter	AFD *n* = 65
Sinus rhythm	63 (96.9%)
Atrial fibrillation	1 (1.5%)
PM rhythm	1 (1.5%)
Heart rate (bpm)	71 [66; 77]
PR interval (ms)	148 ± 21.3
QRS duration (ms)	88 [84; 98]
Right bundle branch block (RBBB)	4 (6.2%)
Left bundle branch block (LBBB)	0 (0%)
LVH by ECG criteria	5 (7.7%)
Max wall thickness (mm)	8 [7; 10.5]
LVMi (g/m^2^)	70 [58; 91]
LVH by echo criteria	11 (17%)
LVEF (%)	65 [61; 66.5]
LV-GLS (%)	−18.5 [−21; −16]
E/e′	6.6 [5.7; 9.3]
LAVi (mL/m^2^)	25 [20; 31]
TAPSE (mm)	22 [20; 31]
TRV-max (m/s)	2.1 ± 0.5
Cardiac Magnetic Resonance performed	13 (20%)
T1 increased	1 (1.5%)
LGE present	9 (13.8%)
LGE extension (>3 segments)	2 (3.1%)

**Table 4:** Summary of Major Adverse Cardiovascular Events (MACE).

Event	*n* (%)
Cardiovascular death	2 (3.1%)
Ischemic stroke (after enrolment)	2 (3.1%)
New-onset atrial fibrillation	2 (3.1%)
NYHA class III–IV progression	1 (1.5%)
Hospitalization for cardiac causes	1 (1.5%)
MINOCA	1 (1.5%)
Ventricular major arrhythmias or ICD therapy	0 (0.0%)
**Total number of patients with at least one MACE**	**12 (18.5%)**
**Total number of MACE**	**25 events**

Note: One additional patient died from a non-cardiovascular cause (work-related trauma).

**Table 5:** Staging Classification in Our Cohort.

Staging System	Stage	*n* (%)
**Meucci**	0	52 (80%)
	1	4 (6.2%)
	2	7 (10.8%)
	3	2 (3.1%)
**Del Franco**	0	27 (41.5%)
	1	3 (4.6%)
	2	8 (12.3%)
	3	27 (41.5%)
**MSSI**	Median score	10.0 [5; 14]
	mild	60 (92.3%)
	moderate	4 (6.2%)
	severe	1 (1.5%)

**Table 6:** Logistic regression results for MACE risk by variant class.

Variant Class	OR	95% CI	*p*-Value
Classical	4.71	1.125; 17.74	0.022
Late-Onset	0.44	0.11; 1.80	0.256
VUS	0.66	0.18; 2.41	0.526

For abbreviations: see the text.

**Table 7:** Association between genotype variant class and clinical staging systems.

Staging	Variant	Coeff.	95% CI	*p*-Value
ME-Stad	CL	3.795	1.052; 13.684	0.041
DF-Stad	CL	3.835	1.169; 12.578	0.027
MSSI	CL	β +9.11	4.08; 14.15	0.0008
ME-Stad	LO	0.705	0.192; 2.586	0.598
DF-Stad	LO	0.862	0.330; 2.252	0.762
MSSI	LO	β −3.75	−8.39; 0.89	0.118
ME-Stad	VUS	0.394	0.097; 1.602	0.193
DF-Stad	VUS	0.445	0.170; 1.162	0.098
MSSI	VUS	β −2.88	−7.53; 1.76	0.229

Results of regression analyses assessing the relationship between genotype class (Classical, Late-Onset, VUS) and three staging systems of cardiac involvement. Ordinal logistic regression was applied for ME-Stad (Meucci, 0–3) and DF-Stad (Del Franco, 0–3), reporting odds ratios (OR) with 95% confidence intervals (CI). Linear regression was used for the MSSI score (continuous), reporting β coefficients with 95% CIs. Classical variants were significantly associated with more advanced stages in both ME-Stad and DF-Stad, and with higher MSSI scores, whereas Late-Onset and VUS variants did not show significant associations. For abbreviations: see the text.

**Table 8:** Sensitivity analysis restricted to patients with available CMR data (*n* = 13).

Genotype	*n*	DF	ME	MSSI	MACE (%)	MACE	LGE (%)
Classical	8	2.5	0.75	21.9	50.0	3.25	62.0
Late-onset	3	2.6	1.33	16.0	33.0	2.0	67.0
VUS	2	2.0	0.0	15.0	50.0	2.0	100.0

## Data Availability

The data presented in this study are available on request from the corresponding author.

## Abbreviations

The following abbreviations are used in this manuscript:

AF	Atrial Fibrillation
AFD	Anderson-Fabry disease
CMR	Cardiac Magnetic Resonance
*GLA*	Alpha-Galactosidase
DF-Stad	Del Franco Stadiation
ERT	Enzyme Replacement Therapy
GLS	Global longitudinal strain
LGE	Late gadolinium enhancement
LVH	Left Ventricle Hypertrophy
MACE	Major Adverse Cardiovascular Event(s)
ME-Stad	Meucci Stadiation
MSSI	Meinz Severity
NSVT	Non-sustained Ventricular Tachycardia
VUS	Variant of unknown significance
